# Impact of the COVID-19 Pandemic on Health-Related Quality of Life, Anxiety, and Training Among Young Gastroenterologists in Romania

**DOI:** 10.3389/fpsyg.2020.579177

**Published:** 2020-12-23

**Authors:** Bogdan Silviu Ungureanu, Catalina Vladut, Felix Bende, Vasile Sandru, Cristina Tocia, Razvan-Aurelian Turcu-Stiolica, Andrei Groza, Gheorghe G. Balan, Adina Turcu-Stiolica

**Affiliations:** ^1^Research Center of Gastroenterology and Hepatology, University of Medicine and Pharmacy of Craiova, Craiova, Romania; ^2^Clinical Emergency Hospital “Prof. Dr. Agrippa Ionescu,” Bucharest, Romania; ^3^Department of Gastroenterology, University of Medicine and Pharmacy “Victor Babes,” Timisoara, Romania; ^4^Faculty of Medicine, Ovidius University of Constanta, Constanta, Romania; ^5^Trueman Consulting, Craiova, Romania; ^6^Prof. Octavian Fodor Regional Institute of Gastroenterology and Hepatology, Cluj-Napoca, Romania; ^7^Department of Gastroenterology, Faculty of Medicine, “Grigore T. Popa” University of Medicine and Pharmacy, Iasi, Romania; ^8^Department of Pharmacoeconomics, University of Medicine and Pharmacy of Craiova, Craiova, Romania

**Keywords:** COVID-19, young gastroenterologists, anxiety, EMAS, quality of life, 15D instrument

## Abstract

The novel COVID-19 infection has spread all over the world and is still generating a lot of issues at different levels. There is a lack of control in disease early diagnosis and rapid evolution, which impacts both the medical and the economic system. Young gastroenterologists should adapt to overcome current difficulties and continue their life and general training. This is a multi-center national study, which aims to assess the general perspective of young gastroenterologists (residents and young specialists) from six university centers in Romania regarding their current training and the psychological effect the pandemic has on their life and job. An online survey with 58 items was distributed using Google Forms, and quality of life and anxiety were assessed. The validated instruments 15D (for assessing the health-related quality of life) and endler multidimensional anxiety scales (EMAS—for assessing anxiety) were used. All analyses were performed using SPSS 25. Of the 174 gastroenterologists approached, 96 (response rate of 55%) responded. A majority of the respondents were residents in gastroenterology (64%), and 40.6% were male. The pandemic influenced the number of examined patients as well as young gastroenterologists’ endoscopy training. Health-related quality of life was negatively associated with the level of anxiety generated by the cognitive component of anxiety as a state, the new and ambiguity of the state, and how threatened the respondent felt. The level of anxiety was moderate (median = 51), and no difference was found between the physicians working in a designated hospital or not. General caution should be considered for young gastroenterologists’ training, and continuous observation should be done to ensure better mental health on the current evolution. These findings would need to be verified in larger-sample studies and in different types of specialties.

## Introduction

The outbreak of the new coronavirus disease (COVID-19), originating in Wuhan, China, has overwhelmed all countries over the world and became the top public health emergency nowadays. While the first cases were reported in December 2019, the World Health Organization declared COVID-19 as a pandemic on 11 March 2020 (Mahase, 2020). As of 24 June 2020, 9.3 million confirmed cases of COVID-19 (477,500 deaths) were recorded all over the world, with 24,826 confirmed cases (1,555 deaths) in Romania. The rapid implementation of control measures successfully prevented a wave in the number of COVID-19 cases in Romania (Dascalu, 2020).

The first confirmed case of COVID-19 has been reported in Romania on February 26, 2020. The COVID-19 pandemic is continuously changing the way we live our lives and also has a substantial influence on the medical staff. General measures were taken in Romania on the medical system, starting from reorganizing hospitals in designated and non-designated COVID-19 institutions to medical personnel redeployment in some situations as well as trying to assure their safety measures when treating patients. Since this is an ongoing process with unforeseen outcomes, medical training should be adapted along with the pandemic evolution. While interaction with tutors may not be encouraged due to social distancing, other alternatives are enrolled on a day-to-day basis. The healthcare system suffered on many fronts due to this unprecedented event.

Physicians are facing a high volume of patients with a contagious condition, which leads to high-risk exposure. This evolved into a stressful situation as many medical practitioners became positive for COVID-19. The contamination risk grew exponentially until protective personal equipment (PPE) became available, and the entire medical staff also learnt to use it. Moreover, university hospitals had to take containment measures by canceling or postponing non-emergency procedures. These sudden changes had to be installed also for residents in gastroenterology due to their typical clinical exposure as well as research activities, resident education, and endoscopy training, which is considered as a high-risk procedure. Moreover, young specialists have more clinical responsibilities on decision-making and are faced with a stressful situation. Young specialists were also affected and were facing a stressful situation since they had more clinical responsibilities.

There is a need of high-quality data on the mental health effect of the COVID-19 outbreak across young physicians also. A study shows that Chinese doctors in training are feeling the force of the COVID-19 pandemic, with increased scores for depression and anxiety (Li et al., 2020). Medical personnel had to be relocated and assigned to designated COVID-19 hospitals, a status which could have been perceived as threatening, with potential negative outcomes on personal lives and medical practice even among young practitioners. Our objective was to assess the pandemic impact on gastroenterology fellows and young specialists by an online survey, which included two validated questionnaires (15D and EMAS). The aims of this study were to examine the perception on gastroenterology training and to evaluate the effect of COVID-19 on the health-related quality of life (HRQoL) and anxiety in gastroenterology residents and young specialists during this pandemic.

## Materials and Methods

### Ethical Issues

This research was approved by the Ethics Committee of the University of Medicine and Pharmacy of Craiova (registration no. 27/2020) according to the Declaration of Helsinki and the University Code of Ethics. The ethics committee approved the study protocol, and all physicians provided electronic informed consent starting with the first question of the survey.

### Study Design

The questionnaire, which included 58 items, was developed and distributed using Google Forms. The participants were recruited from the gastroenterology departments from the public hospitals of major university centers in Romania, Bucharest, Craiova, Cluj-Napoca, Constanta, Iasi, and Timisoara (nine public hospitals). The inclusion criteria were as follows: resident or young specialist working in the gastroenterology department. Participants from designated COVID-19 hospitals and non-designated COVID-19 hospitals were enrolled in this survey to compare differences between the two types of hospitals. The survey was conducted from April 21, 2020 to May 9, 2020 at the request of the Young Romanian Gastroenterologists Organization.

The survey was anonymous and confidential. An introductory paragraph outlining the purpose of the study and the protection of respondents with regard to the processing of personal data (Regulation EU 2016/679) was posted along with the survey.

### Outcomes

The questionnaire was structured in four sections. Section 1 had 18 items: five items collected the demographic information of the respondents (age, gender, marital status, year of training, type of hospital, and access to training) and 13 items were designed to evaluate the different aspects of the COVID-19 situation. In particular, the following aspects were evaluated: access to training, PPE, and personal safety procedure. Endoscopic training was evaluated with questions about the number of endoscopic procedures (upper and inferior) before and during the difficult time. Section 2 comprised the 15 questions from the 15D Instrument about HRQoL. Section 3 comprised five items from the EMAS- Perception (EMAS-P) questionnaire. Section 4 comprised 20 items from the EMAS-State (EMAS-S).

The physicians were grouped, according to their training level, into two groups: gastroenterology fellows group and young specialist group, and the outcomes were compared.

The respondents were also grouped, according to the hospital where they work, into two groups: designated hospital and non-designated hospital, and the outcomes were compared.

#### 15D Instrument

The 15D instrument is a generic, multidimensional, self-administered evaluative tool for assessing HRQoL, with 15 dimensions: mobility, vision, hearing, breathing, sleeping, eating, speech, excretion, usual activities, mental function, discomfort and symptoms, depression, distress, vitality, and sexual activity (Sintonen, 2001). The Romanian language version of the 15D was used (Subtirelu et al., 2019; Padureanu et al., 2020). The single score (15D score) was calculated representing the overall HRQoL on a 0 to 1 scale, where 0 = being dead and 1 = full health.

#### EMAS

Endler multidimensional anxiety scale is an instrument that measures the state and trait anxiety in people with and without anxiety symptoms (Endler et al., 1991). We administered the Romanian validated EMAS (Miclea et al., 2002), performing EMAS-S with 20 items and EMAS-P with five items. EMAS-S measures state anxiety in relation to autonomic–emotional (AE) and cognitive–worry (CW) components. The EMAS-P of the situation (COVID-19 in our study) is a measure of the subjective perception of the type of situation (that is, COVID-19) and the degree of threat evoked by this particular situation as experienced by the individual at the time of testing. EMAS-P gave five different scores: EMAS-P-ES (the scale evaluates the extent to which the respondent perceives the situation at the time of testing as a situation of social evaluation), EMAS-P-PF (the scale assesses the extent to which the respondent perceives the situation at the time of testing as a situation of physical danger), EMAS-P-AM (the scale evaluates the extent to which the respondent perceives the situation at the time of testing as a new and ambiguous situation), EMAS-P-RZ (the scale evaluates the extent to which the respondent perceives the situation at the time of testing as a daily routine situation), and EMAS-P-A (the scale assesses how threatened the respondent felt in the situation at the time of testing). Assessing the type and the intensity of the perceived threat, as measured by EMAS-P, is also important for understanding the respondent’s specific pattern of anxiety responses. All EMAS scores were converted to standard T points from 0 to 100. The medium values are considered to be between 40 and 60. High scores indicate a high level of anxiety.

### Statistical Analysis

Descriptive statistics and percentages were used to summarize the data. Continuous data are expressed as mean ± SD (for normally distributed variables) or median (interquartile range, for not normally distributed variables). Investigation of histograms and the Shapiro–Wilk test revealed if the continuous variables were normally distributed. When the variable was continuous, comparisons between two groups were performed using *t-*test (if normally distributed) or Mann–Whitney *U* test (if not normally distributed). We assessed the differences between residents vs. young specialists, designated hospital vs. non-designated hospital, and activities before COVID-19 vs. activities in the time of COVID-19. When the variable was categorical, *χ*^2^ test was used. The correlation matrix was analyzed for assessing the significant correlations between HRQoL and anxiety scores. Spearman correlation coefficient was used in case of lack of normality in data. Statistical analysis was performed using SPSS software, version 25 (IBM SPSS, Armonk, NY, United States). A *p*-value < 0.05 was considered as statistically significant.

## Results

Of the 174 young gastroenterologists working at the time of the pandemic in the nine public hospitals, only 96 (response rate of 55%) have responded to our survey. The median time taken to complete the survey was 5.0 min. Among the respondents, 39 (40.6%) were male, and 64 (66.7%) were gastroenterology fellows. The average age was 29 years (SD = 3.27), with a range of 24–38 years. There were more females than males in the sample (59.4 vs. 40.6%). More than a half were not married (61.5%). Table 1 shows the distribution of the survey participants by socio-demographics and their responses to the questions related to their activity before and in the time of COVID-19.

**TABLE 1:** Demographics and initial answers of the survey participants.

Characteristics	Category	Number (percentages)
Age	24 25–29 30–34 35–39	2 (2%) 60 (63%) 26 (27%) 7 (7%)
Gender	Female Male	57 (59.4%) 39 (40.6%)
Marital status	Married Not married Divorced	33 (34.4%) 59 (61.5%) 3 (3.1%)
Medical training	Residents Specialists	64 (66.7%) 32 (33.3%)
COVID-19 designated hospital	Yes No	25 (26%) 71 (74%)
Do you have colleagues infected with COVID-19?	Yes No	19 (19.8%) 77 (80.2%)
Do you think the pandemic influenced the training?	Yes No	90 (93.8%) 6 (6.2%)
Do you think that your coordinators were less involved in the training?	Yes No	48 (50%) 48 (50%)
Access to the PPE	Yes No	55 (57.3%) 41 (42.7%)
Knowing how to use the PPE	Yes No	76 (79.2%) 20 (20.8%)

*PPE, protective personal equipment.*

We asked the respondents to rate their activity during the outbreak, and 26% of them were practicing or have been redirected to COVID-19 patient-dedicated hospitals. Most of the residents (98%) stated that the COVID-19 outbreak did influence their status on gastroenterology training. The participants indicated that 57.3% had access to PPE and 79.2% know how to use the PPE. In terms of knowing the safety procedures in the workplace, 76 respondents responded that they had known of such, of which 48 were residents, without significant differences between residents and specialists (*p* = 0.2). However, 19.8% of them confirmed that they had infected colleagues.

When asked about their perception over medical training, they suggested that nearly half (49.5%) of the residency or clinic coordinators were less involved in their medical development and their apprenticeship.

The pandemic also influenced the number of patients examined by each physician as well as their endoscopy training (Table 2). The number of patients and of endoscopies per month was assessed two times: before the World Health Organization declared the COVID-19 pandemic on March 2020 (before COVID-19) and upon applying the survey from April 21, 2020 to May 9, 2020 (now). We used the corresponding dates of the year 2019 for the period before COVID-19.

**TABLE 2:** Characteristics of physicians for the two groups.

Characteristics	Gastroenterology fellow group (*n* = 64)	Young specialist group (*n* = 32)	*p*-value
Gender, male^∧^	22 (34%)	17 (53%)	0.078
Age, years*	27 (2)	31 (3)	<0.01
Patients before COVID-19, number/month*	40 (40)	60 (110)	<0.05
Patients now, number/month*	15 (18)	20 (35)	0.077
Upper endoscopy before COVID-19, number/month*	10 (25)	30 (38)	<0.05
Upper endoscopy now, number/month*	0 (2)	5 (10)	<0.05
Colonoscopy before COVID-19, number/month*	5 (40)	20 (20)	<0.05
Colonoscopy now, number/month*	0 (0)	2 (5)	0.065
Influence, yes^∧^	63 (98%)	27 (84%)	0.015
Less involved, yes^∧^	37 (58%)	10 (31%)	0.035
Equipment, yes^∧^	33 (52%)	22 (69%)	0.129
Correct use, yes^∧^	48 (75%)	28 (88%)	0.298
HRQoL*	0.966 (0.055)	0.966 (0.036)	0.116
EMAS-S-AE*	50 (17)	50 (31)	0.562
EMAS-S-CW*	53 (17)	50 (9)	0.058
EMAS-S-T*	52 (17)	50 (10)	0.176
EMAS-P-ES*	54 (10)	46 (11)	0.078
EMAS-P-PF*	42 (7)	47 (7)	0.497
EMAS-P-AM*	61.5 (18)	55 (9)	0.304
EMAS-P-RZ*	42 (15)	43 (12)	0.875
EMAS-P-A*	52 (14)	58.5 (18)	0.728

*median (interquartile range); ^∧^*n* (%); HRQoL, health-related quality of life; EMAS-S-AE, EMAS state from the autonomic–emotional component; EMAS-S-CW, EMAS state from the cognitive–worry component; EMAS-S-T, EMAS state total; EMAS-P, EMAS perception; ES, social evaluation threat perception; PF, physical danger threat perception; AM, ambiguous threat perception; RZ, daily routines threat perception; A, threat situation.

Investigation of normality for continuous variables revealed a significant difference in the sample distribution from the normal distribution. We used non-parametric statistics to describe the results of the study due to these issues.

The highest values for anxiety were found for EMAS-P-AM, higher than the medium values, with an average value of 57.50 (±10.2), without significant differences between residents and specialists.

Women did not present more intense state anxiety than men (EMAS-S-T score 53 vs. 50, *p* = 0.319), but they present more ambiguity anxiety than men (EMAS-P-AM score 61 vs. 53, *p* = 0.011). The physicians from non-designated hospitals believed more than the physicians from designated hospitals that involvement was less in training, but not statistically significant (*p* = 0.07). As Table 3 shows, there are small differences between the analyzed characteristics from the designated hospital vs. those from the non-designated hospital.

**TABLE 3:** Evaluation of characteristics from designated hospitals vs. non-designated hospitals.

Designated hospital (*n* = 25)		Non-designated hospital (*n* = 71)	*p*-value
Age*	28 (2)	28 (6)	0.395
Influence, yes^∧^	23 (92%)	67 (94%)	0.65
Less involved, yes^∧^	17 (68%)	30 (42%)	0.07
HRQoL*	0.957 (0.061)	0.966 (0.041)	0.888
EMAS-S-EF*	54 (15)	49 (13)	0.195
EMAS-S-CW*	52 (13)	51 (13)	0.352
EMAS-S-T*	53 (15)	50 (12)	0.406
EMAS-P-ES*	54 (10)	52 (10)	0.9
EMAS-P-PF*	42 (12)	47 (7)	0.948
EMAS-P-AM*	62 (15)	61 (18)	0.406
EMAS-P-RZ*	44 (13)	42 (14)	0.808
EMAS-P-A*	61 (20)	52 (16)	0.035

*median (interquartile range); ^∧^*n* (%); HRQoL, health-related quality of life; EMAS-S-AE, EMAS state from the autonomic–emotional component; EMAS-S-CW, EMAS state from the cognitive–worry component; EMAS-S-T, EMAS state total; EMAS-P, EMAS perception; ES, social evaluation threat perception; PF, physical danger threat perception; AM, ambiguous threat perception; RZ, daily routines threat perception; A, threat situation.

**FIGURE 1:**
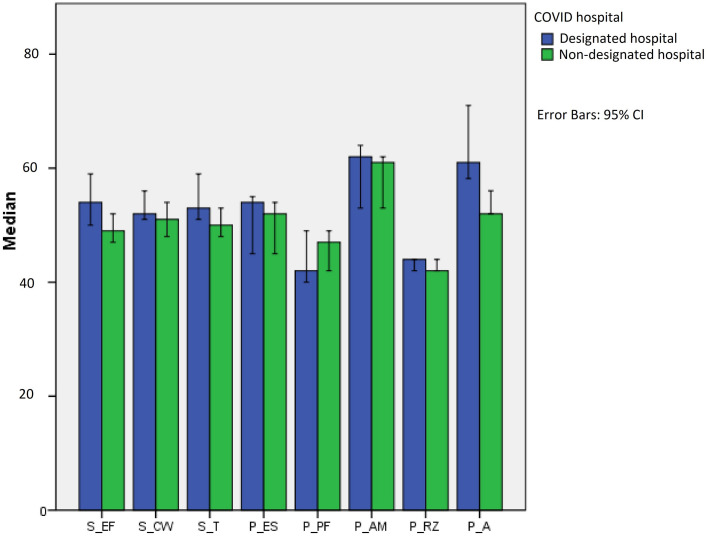
The endler multidimensional anxiety scales anxiety scores’ differences between the designated hospitals and non-the designated hospitals.

The level of anxiety was not different between the physicians working in a COVID-19 hospital or not (*p* > 0.05, for EMAS-S-T). The component of anxiety EMAS-P-A (perception of threat situation) scores in the designated hospital group was higher than normal and, compared with the non-designated hospital group, the difference was statistically significant (*p* < 0.05), as shown in Figure 1. The physicians had the same moderate anxiety being involved in their daily routines (*p* > 0.05, for EMAS-P-RZ) and the same higher anxiety being in a new and ambiguous situation (*p* > 0.05, for EMAS-P-AM).

The number of upper and inferior endoscopies was less performed in the time of COVID-19 than before COVID-19 (Table 4).

**TABLE 4:** Characteristics of activities before and in the time of COVID-19.

Before COVID-19 Mean (SD)		Now Mean (SD)	*p*-value
Patients*	69.9 (77.5)	25.4 (34.5)	<0.001
Upper endoscopy*	23.1 (22)	3.4 (6.2)	<0.001
Colonoscopy*	12.9 (15.1)	2 (5.3)	0.001

*number per month.

HRQoL was negatively associated with the level of anxiety generated by the cognitive component of anxiety as a state (S-CW, S-T), the ambiguity of the state (P-AM), and how threatened the respondent felt (P-A) (see Table 5 for the complete correlation matrix).

**TABLE 5:** Correlations between the variables.

Variable	Age	HRQoL	S-AE	S-CW	S-T	P-ES	P-PF	P-AM	P-RZ	P-A
Age	1	0.12	0.03	−0.12	−0.07	−0.2	0.11	−0.11	−0.02	0.02
HRQoL		1	−0.15	−0.34**	−0.28**	−0.17	−0.04	−0.27**	0.22	−0.28**
S-AE			1	0.75**	0.93**	0.3**	0.44**	0.33**	−0.07	0.31**
S-CW				1	0.93**	0.35**	0.26*	0.35**	−0.09	0.32**
S-T					1	0.33**	0.35**	0.36**	−0.09	0.32**
P-ES						1	0.22*	0.28**	−0.07	0.41**
P-PF							1	0.15	−0.02	0.25*
P-AM								1	−0.34**	0.44**
P-RZ									1	−0.15
P-A										1

**correlation is significant at the 0.01 level; *correlation is significant at the 0.05 level; HRQoL, health-related quality of life; S-AE, EMAS state from the autonomic–emotional component; S-CW, EMAS state from the cognitive–worry component; S-T, EMAS state total; P-ES, social evaluation threat perception; P-PF, physical danger threat perception; P-AM, ambiguous threat perception; P-RZ, daily routines threat perception; P-A, threat situation.

## Discussion

Focusing on fellows and young specialists in gastroenterology in Romania, our findings illustrate their very good level of HRQoL (the value is higher than 0.95) in the first wave of the COVID-19 pandemic, with no differences between designated hospitals and non-designated hospitals. Health-related quality of life was negatively associated with the level of anxiety generated by the cognitive component of anxiety as a state, the new and ambiguity of the state, and how threatened the respondents felt. However, the pandemic had a major impact on a psychosocial level. Understanding the impact of the COVID-19 outbreak is crucial in the development of policy guidelines and interventions for future possible pandemics. We aimed to assess the anxiety of young gastroenterologists within the COVID-19 outbreak. The highest values for anxiety were the result of the new and the ambiguity of this period. The healthcare workers’ lives were surrounded by fear during COVID-19 pandemic’s phase 1 (Marton et al., 2020). The disease’s frequent information changes generate fear and worry, a fact previously reported in prior outbreaks in 2003 in the case of severe acute respiratory syndrome (SARS) (Rambaldini et al., 2005). Shad et al. (2020) have suggested approaches in managing these challenges.

In another study exploring the psychological impact of SARS outbreak on physicians, younger doctors were more likely to have high posttraumatic stress symptoms associated with fear of SARS outbreak (Wu et al., 2009). Xiao et al. (2020) found that the stress level of young doctors during COVID-19 was higher than that during SARS.

The anxiety of physicians in the COVID-19 outbreak was also assessed by Wu and Wei (2020). They obtained the same moderate anxiety, without differences between physicians working at designated hospitals vs. non-designated hospitals, which is similar to our results. In this stressful COVID-19 outbreak, ambiguous was the dimension of trait anxiety which increased the total level of anxiety. All the physicians are facing a fast, new, moving, and ambiguous situation, with increasingly difficult-to-face challenges (Barello et al., 2020).

We found that being a woman was not associated with lower or higher anxiety than being a man, unlike other studies that have found that being younger and a woman, having less professional experience, and working in the frontline were associated with higher scores of anxiety (Elbay et al., 2020). Differences were found for perception of ambiguity when women presented more ambiguity anxiety than men.

The existing evidence of anxiety among healthcare workers was already done using random-effects meta-analysis (Pappa et al., 2020). The pooled prevalence rate of anxiety was found to be 23.2%, with female respondents exhibiting higher rates of anxiety compared to male respondents. A subgroup analysis with age criteria or with numbers of confirmed cases of COVID-19 per country should also be done. Giusti et al. (2020) found that 71.2% of health professionals working in Northern Italy had scores of state anxiety above the clinical cutoff in the COVID-19 pandemic period.

The mental well-being of all medical healthcare providers is still at stake, as some of them have been in the situation of treating their colleagues or face the fact that they may transmit the infection to their siblings. Our findings also suggest that the pandemic effect has an impact on their work quality on a daily basis, regardless of treating COVID-19-infected patients. All fellows in training should perform and be present in a number of endoscopic procedures, but during the pandemic this goal might not be achieved due to all general recommendations of limiting the interventions. Noteworthy are also the long-term effects that the pandemic will cause since many patients will delay their clinical visits and how the medical system will reboot.

While only a quarter of the participants were working in designated COVID-19 hospitals, more than half of them were still supposed to continue their medical training. This became a problem all over the world and not only for medical faculties since all teaching programs became affected. Rotations for all medical staff started in all hospitals, even for fellows in gastroenterology. This made it difficult for them to interact with patients as well as to participate in endoscopic procedures. The restrictions were instated along with national emergency status, and the need for new training and teaching methods became necessary. The pandemic clearly affected their daily practice, with a very low rate of patients and endoscopic procedures daily. However, this encouraged telemedicine to step forward and draw them in new methods of interactions.

The gastroenterology fellows are well aware of e-learning and most of the available platforms that may improve their general training. However, this type of interaction should be more engaged in this period. Given the current situation, most of them are unsure of their medical evolution and their career development. This gap might be filled for now by the use of technology and also by a reorganization of the tutor’s way of teaching. Moreover, 50% of the participants stated that their coordinator was not as involved as previously. Also, there was a lack of participation in and performing endoscopic procedures since a general decrease came along with the patients’ admissions at the start of the pandemic. For endoscopy trainees, the lack of procedures represents the most important aspect, as they are required a specific number to complete their training and also to become proficient. This raises the question of whether their training period should be extended.

Our study included both fellows and young specialists because they represent two important steps in gastroenterology, and the pandemic could affect them at different levels. While fellows are eager to learn and improve their knowledge, which are now limited by the pandemic, the young specialists who are just beginning to develop their doctor life are confronted with a situation for which they were not trained during their residency and now have more responsibilities.

Questions are also to be answered as to how to restart gastroenterology training from this point on. While the focus will generally be on testing and access to PPE, endoscopy procedures will still need to be balanced and still assure high-quality training for young practitioners. Fellows should be well instructed on infection control and proper PPE use. Our survey revealed that only 57% had proper PPE equipment, which suggests that this might also have an emotional impact on some of them as they may not feel safe. Moreover, the use of PPE becomes even more stressful in the endoscopy rooms as there are procedures with potential contamination risks.

Senior fellows may face another issue as their graduation is at stake, and after finishing their fellowship, they may not feel as prepared as they should be since this period changed the healthcare system. Thus, their coordinators should help them focus on available telemedicine methods, enhance their communication skills, and interact with other graduates so that this transition may be easier. Currently, gastrointestinal societies are encouraging online communication methods by different scheduled meetings and webinars and even try to keep their international congresses by broadcasting experts from their institution (Shad et al., 2020).

This study has several limitations. First, most participants (73.96%) were from non-designated hospitals. Romania has not been hit by the virus as hard as the other countries, and not too many residents in gastroenterology work in designated hospitals. Second, while the number of participants that answered the survey is not large, we believe that our results are relevant for the developments that are currently taking place in Romania. Third, the survey lasted 21 days and lacks longitudinal follow-up, but it caught the peak period and we used Romanian validated questionnaires.

Young gastroenterologists remain as some of the exposed part of the medical staff, especially with the need of performing endoscopies. The levels of distress (anxiety) in the time of COVID-19 are encouraging, though it should be monitored for a longer period according to the pandemic evolution.

In conclusion, the COVID-19 pandemic provides a major uncertainty for young gastroenterology practitioners. General caution should be considered for their current medical practice, and more attention should be focused on their training using technology since other methods are unavailable at this moment. We found a moderate level of anxiety during the first wave of COVID-19 pandemic in Romania among them, and we considered that a continuous observation should be done from different national institutions to provide a better psychological follow-up on the current developments.

## Data Availability Statement

The raw data supporting the conclusions of this article will be made available by the authors, without undue reservation.

## Ethics Statement

The studies involving human participants were reviewed and approved by Ethics Committee of the University of Medicine and Pharmacy of Craiova. The patients/participants provided their online informed consent to participate in this study.

## Author Contributions

BU, CV, FB, VS, CT, AG, and GB: data curation. BU, R-AT-S, and AT-S: formal analysis. CT: investigation. R-AT-S and AT-S: methodology. BU and CT: project administration. R-AT-S: resources. BU: supervision. CT: visualization. BU and AT-S: writing – original draft, writing – review, and editing. All authors contributed to the article and approved the submitted version.

## Conflict of Interest

R-AT-S was employed by the company Trueman Consulting. The remaining authors declare that the research was conducted in the absence of any commercial or financial relationships that could be construed as a potential conflict of interest.
